# Real and synthetic data sets for benchmarking key-value stores focusing on various data types and sizes

**DOI:** 10.1016/j.dib.2020.105441

**Published:** 2020-03-20

**Authors:** Hyuk-Yoon Kwon

**Affiliations:** ITM Division, Dept. of Industrial Engineering, Seoul National University of Science and Technology, 232 Gongneung-Ro, Nowon-Gu, Seoul 01811, South Korea

**Keywords:** Key-value pairs, Key-value stores, Big data, Geographic location, Social network analysis, Twitter, Benchmark

## Abstract

In this article, we present real and synthetic data sets for benchmarking key-values stores. Here, we focus on various data types and sizes. Key-value pairs in key-value data sets consist of the key and the value. We can construct any kinds of data as key-value data sets by assigning an arbitrary type of data as the value and a unique ID as the key. Therefore, key-value pairs are quite worthy when we deal with big data because the data types in the big data application become more various and, even sometimes, they are not known or determined. In this article, we crawl four kinds of real data sets by varying the type of data sets (i.e., variety) and generate four kinds of synthetic data sets by varying the size of data sets (i.e., volume). For real data sets, we crawl data sets with various data types from Twitter, i.e., Tweets in text, a list of hashtags, geo-location of the tweet, and the number of followers. We also present algorithms for crawling real data sets based on REST APIs and streaming APIs and for generating synthetic data sets. Using those algorithms, we can crawl any key-value pairs of data types supported by Twitter and can generate any size of synthetic data sets by extending them simply. Last, we show that the crawled and generated data sets are actually utilized for the well-known key-value stores such as Level DB of Google, RocksDB of Facebook, and Berkeley DB of Oracle. Actually, the presented real and synthetic data sets have been used for comparing the performance of them. As an example, we present an algorithm of the basic operations for the key-value stores of LevelDB.

Specifications Table

**Table:** 

Subject	Computer Science
Specific subject area	Information Systems, Social Networking Analysis
Type of data	Raw data in text files
How data were acquired	Real data sets are crawled by the Python program, which is shown in Algorithms 1 and 2, and synthetic data sets are generated by the Python program, which is shown in Algorithm 3
Data format	Raw, analysed, descriptive, and statistical data
Parameters for data collection	For real data sets, we crawl them using REST APIs and streaming APIs. For synthetic data sets, we vary the parameter for the size of the data set: 1) 10 K, 2) 100 K, 3) 1 M, and 4) 10 M. The details are explained in Sections 2.1 and 2.2.
Description of data collection	For real data sets, we implement a crawling method from Twitter based on Tweepy [4], which is a Python library for accessing to Twitter APIs. Here, we vary the data types supported by Twitter when we construct the key-value pairs for each data set. For synthetic data sets, we devise a method to generate data sets with various data sets. As a result, the method receives the size of data sets as the input and generates a given size of the data set.
Data source location	Crawled from Web for real data sets; generated for synthetic data sets;
Data accessibility	The data sets are publicly available at Mendeley Data: http://dx.doi.org/10.17632/kxcb3tnr3t.2
Related research article	Hyuk-Yoon Kwon, “Constructing a Lightweight Key-Value Store Based on the Windows Native Features,” Applied Sciences, Vol. 9, No. 18, Article 3801, pp. 1–22, Sept. 2019 (https://doi.org/10.3390/app9183801).

## Value of the Data

• We can construct arbitrary data types appeared in big data applications as key-value pairs because we can map any kinds of data types as the value of key-value pairs. We present real and synthetic data sets for key-value pairs. They include various data types (i.e., data variety) in four real data sets and various sizes of the data sets (i.e., data volume) in four synthetic data sets.
• The synthetic data sets are useful when the variable sizes of data sets are required for benchmarking key-value stores to evaluate their scalability as the size of data sets is increased because the size of the provided data sets is varied from 10 K to 10 M. The real data sets are useful for benchmarking key-value stores to evaluate their performance variation for different data types. We choose Twitter to crawl various data types because Tweets contain multiple data types such as geographic location, hash tags, Tweets, and the number of followers. These real and synthetic data sets were actually used to compare the performance of the well-known key-value stores such as LevelDB of Google [1], RocksDB of Facebook [2], and BerkeleyDB of Oracle [3].
• We present algorithms to crawl real data sets and to generate synthetic data sets. They provide a template to crawl real data sets for various data types that can be crawled from Twitter and to generate synthetic data sets for the various sizes of data sets. We note that the presented algorithms can be easily extended for more various data types and more various sizes of data sets.

## 1 Data description

In this article, we present key-value data sets where each data set is composed of various data types. We present eight datasets including real and synthetic data sets for storing them in the key-value stores such as LevelDB of Google [1], RocksDB of Facebook [2], and Berkeley DB of Oracle [3]. The key-value stores have a strength that can deal with various data types by assigning data of an arbitrary type as the value and the unique ID as the key. That is, the key-value stores can support various data types, i.e., not only primitive data types such as integer and string but also composite data types such as collection and array of primitive data types. When we construct key-value data sets, we focus on various data types (i.e., variety) in real data sets and various sizes of data sets (i.e., volume) in synthetic data sets. These are two important factors for benchmarks of key-value stores to perform their performance evaluation.

In this article, as an example of real data sets for key-value stores, we choose Twitter to crawl various data types because Tweets contain multiple data types such as geographic location, hash tags, Tweets, and the number of followers. Table 1 shows an example data type supported by Twitter.^1^ As shown in Table 1, there are many types of data in a Tweet. Thus, it could be a suitable candidate of data sets for benchmarking key-value stores.

**Table 1:** An example data type in Twitter.

Attributes	Description	Data types	Data examples
ID	Unique identifier for Tweet	Int64	1,050,118,621,198,921,728
Text	Actual text of the status update	String	“To make room for more expression, we will now count all emojis as equal—including those with gender and skin t… https://t.co/MkGjXf9aXm”
Truncated	Indicates whether the value of the text attribute was truncated	Boolean	true
Coordinates	The longitude and latitude of the Tweet's location	Collection of Float	[−97.51087576,35.46500176]
created_at	UTC when the Tweet is created	String	“Wed Oct 10 20:19:24 + 0000 2018”
Urls	URLs included in the text of a Tweet	Array of URL Objects	“url”: “http://t.co/IOwBrTZR”, “display_url”: “youtube.com/watch?v=oHg5SJ…”, “expanded_url”: “http://www.youtube.com/watch?v=oHg5SJYRHA0”

As an example of key-value data sets, we vary data types supported by Twitter. That is, all the data sets are designed to have different data types such as Integer, String, Boolean, and Collection. Specifically, we use Tweet ID or User ID as the key part; we use text, Coordinates, hashtags, or the number of followers as the value part. Table 2 shows characteristics of the real data sets. We crawled four kinds of real data sets: (1) ID-Geo, consisting of the tweet ID and the location information of the tweet, (2) ID-Hashtag, consisting of the tweet ID and the hash tags in the tweet, (3) ID-Tweet, consisting of the tweet ID and the tweet text, and (4) User-Followers, consisting of the user ID and the number of followers of the user. Table 3 shows the samples of real data sets. As presented, we can check that various data types are used as the value of the key-value data set. The detailed algorithm to crawl these data sets will be explained in Section 2.1.

**Table 2:** Characteristics of the real data sets.

Data Sets	Total Number of Objects	Average of Key Size	Average of Value Size	Total Size of Data Set	Data Types
ID-Geo	2656,660	19.00	28.34	122MB	Key: Int64, Value: Collection of float
ID-Hashtag	172,958	19.00	25.31	7.4MB	Key: Int64, Value: String
ID-Tweet	1556,300	19.00	135.54	234MB	Key: Int64, Value: String
User-Followers	140,438	12.27	5.06	2.5MB	Key: Int64, Value: Int

**Table 3:** The samples of real data sets.

Data Sets	Key	Value
ID-Geo	1,020,235,969,885,134,848	(−115.223125, 36.232915)
ID-Hashtag	1,020,253,494,912,135,168	#OrangeCounty #I4 #Orlando #traffic
ID-Tweet	1,020,315,941,345,923,074	A Chocolate man with a pretty smile
User-Followers	429,997,348	1016

Table 4 shows characteristics of the synthetic data sets. In synthetic data sets, we focus on the size of data sets. We generate four synthetic data sets according to the various size of data set: (1) KVData1, (2) KVData2, (3) KVData3, and (4) KVData4. The total number of objects are varied from 10 K to 10 M. For the data type of each key-value pair, we fix an integer as the key and a string as the value so that we can focus on the size of data sets. The detailed algorithm to generate these data sets will be explained in Section 2.2.

**Table 4:** Characteristics of the synthetic data sets.

Data Sets	Total Number of Objects	Average of Key Size	Average of Value Size	Total Size of Data Set	Data Types
KVData1	10,000	3.89	52.44	560KB	Key: Int, Value: String
KVData2	100,000	4.89	52.53	5.7MB	Key: Int, Value: String
KVData3	1,000,000	5.89	52.54	58MB	Key: Int, Value: String
KVData4	10,000,000	6.89	52.50	589MB	Key: Int, Value: String

## 2 Experimental design, materials, and methods

### 2.1 A method for crawling real key-value data sets

For real key-value data sets, we crawl various data types from Twitter. Twitter provides two kinds of APIs to crawl the Tweets: REST APIs and streaming APIs.^2^ REST APIs and streaming APIs work in a completely different way. The former allows to crawl Tweets filtered by search conditions from Twitter; The latter allows to crawl real time data by sending requests to Twitter continuously. For crawling real data sets from Twitter, we also use two kinds of algorithms where one uses REST APIs and the other uses streaming APIs. For both of them, we use Tweepy [4] that is a Python library to support mapper APIs for using the Twitter APIs. In Table 2, we present four kinds of real data sets crawled from Twitter. Three of them are crawled by using REST APIs; one of them by streaming APIs.

**Fig. 1:**
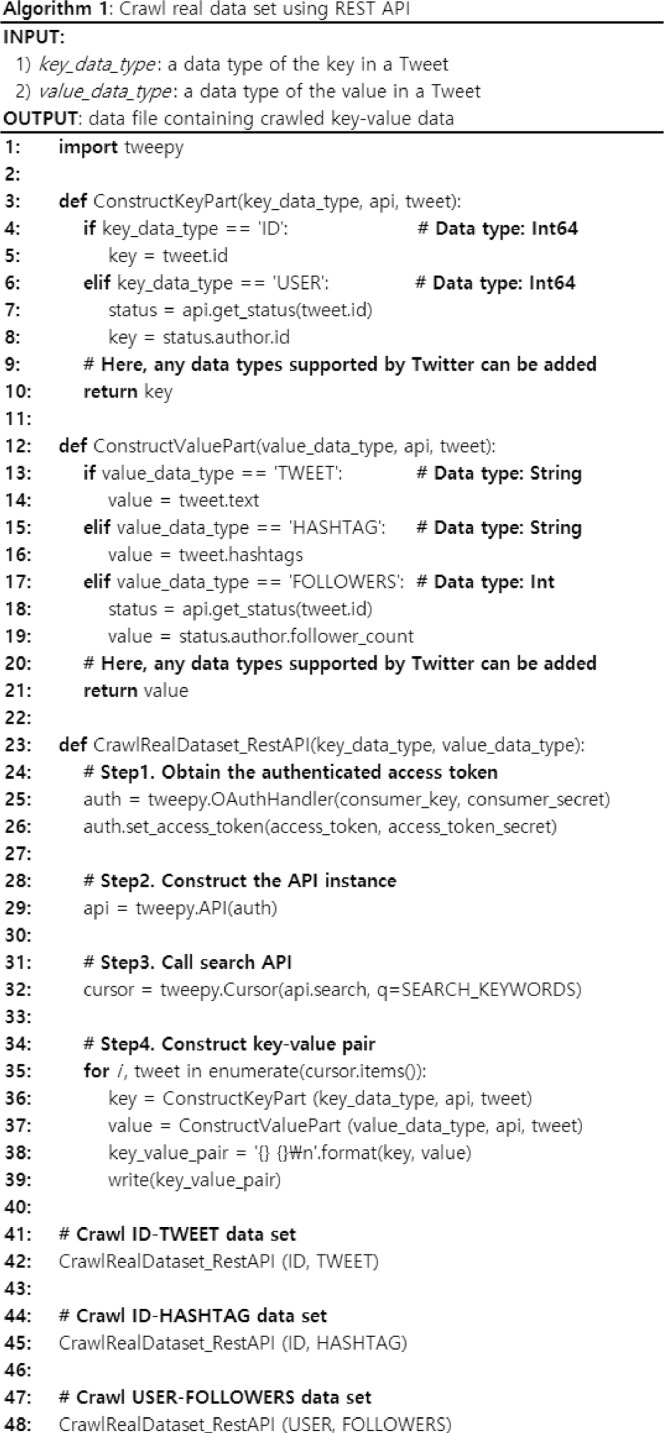
The algorithm for crawling real data sets using REST API.

Fig. 1 shows an algorithm that crawls real data sets using REST APIs. Here, we focus on crawling of various key-value data types. Thus, as examples of real data set, we use specific data types presented in Table 2. However, we can easily extend them to other data types supported by Twitter. As a result, the input of the algorithm is 1) *key_data_type*, a data type of the key in a Tweet, and 2) *value_data_type,* a data type of the value in a Tweet. The output of the algorithm is a data file containing crawled key-value data. In the algorithm, it first obtains the authenticated access token via OAuth authentication. Next, it constructs the API instance using the authenticated access token. Then, it calls the search API with a parameter: *keywords*, a list of search keywords for filtering Tweets.^3^ For the data sets in Table 2, we use the most general keyword “the” to crawl all the normal Tweets. Last, it enumerates each item and constructs key-value pair data for a given data type. Here, we define *ConstructKeyPart*() and *ConstructValuePart*() to support multiple data types for the key and value, respectively. Currently, each function defines cases for the data types required in Table 2. However, we can easily add other pairs of data types supported by Twitter by defining them in a similar way. By calling the function with different data types for the key and value, we crawl three kinds of different key-value data types that can be crawled from Twitter: 1) ID-TWEET (i.e., a pair of the tweet ID and a tweet in text), 2) ID-HASHTAG (i.e., a pair of the tweet ID and a list of hashtags in the tweet), and 3) USER-FOLLOWERS (i.e., a pair of the user ID and the number of followers to the user).

**Fig. 2:**
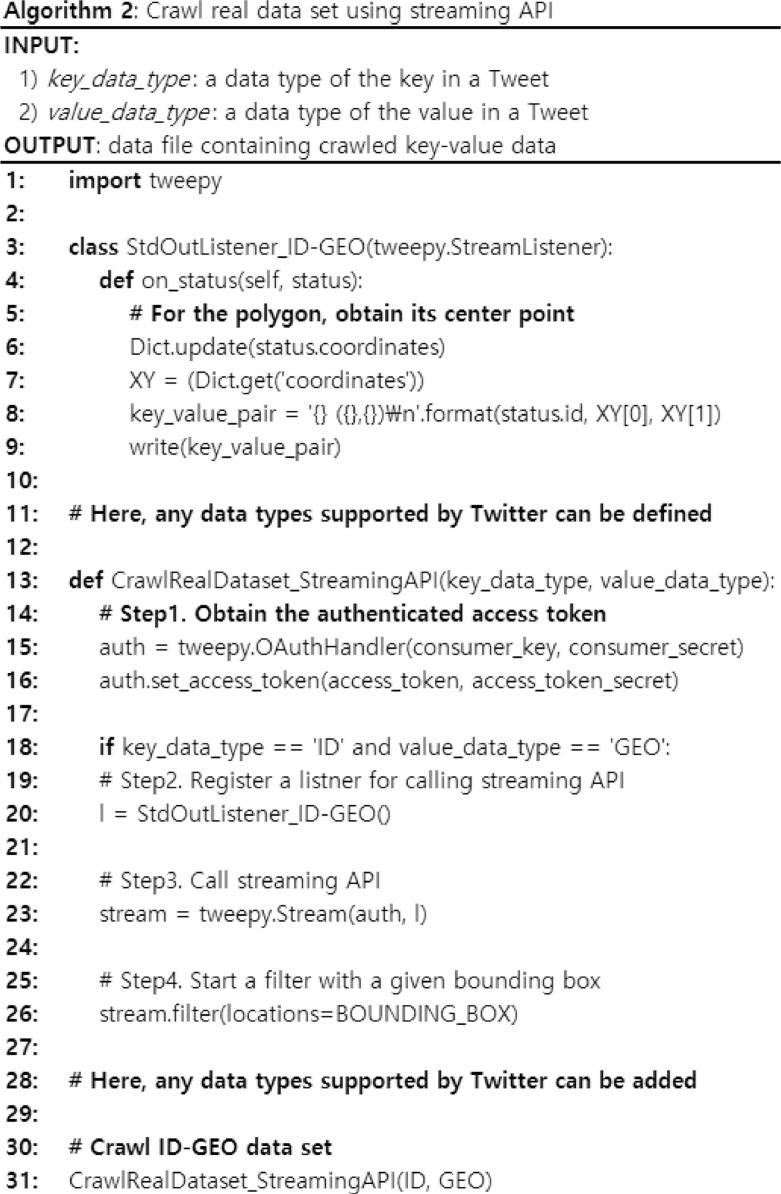
The algorithm for crawling real data sets using streaming API.

Fig. 2 shows an algorithm that crawls real data sets using streaming APIs. The input of the algorithm is 1) *key_data_type*, a data type of the key in a Tweet, and 2) *value_data_type,* a data type of the value in a Tweet. The output of the algorithm is a data file containing crawled key-value data. Here, we crawl a data set, ID-GEO (i.e., a pair of the ID and geo location), but we can extend this algorithm into any key-value pairs of other data types supported by Twitter by defining classes for other data types in a similar way. In the algorithm, it first obtains the authenticated access token as the same way in Algorithm 1. Next, it registers a listener for calling the streaming API. Then, it calls the streaming API. Here, we use the bounding box for covering the region of USA for filtering Tweets. Last, it starts a filter with a given bounding box. For every real time Tweet satisfies the filter, the registered listener will act. In the listener, we extract (x, y) coordinates of Tweets and construct a key-value pair using the Tweet ID as the key and the extracted (x, y) coordinates as the value.

### 2.2 A method for generating synthetic key-value data sets

**Fig. 3:**
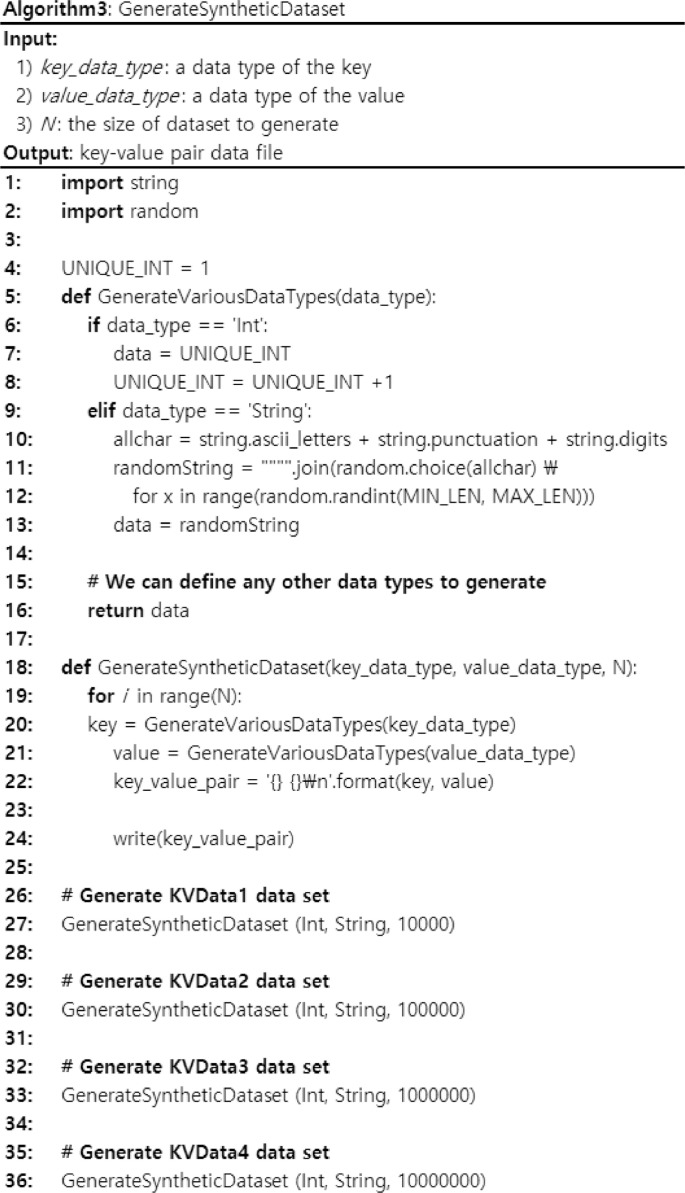
The algorithm for generating synthetic data sets.

Fig. 3 shows an algorithm that generates synthetic data sets. The main purpose of the algorithm generates a synthetic data set by varying the size of data set. Here, we fix an integer as the key and a string as the value to focus on various sizes of data sets. However, the other data types can be easily supported by defining them as separate cases in a similar way. The input of the algorithm is as follows: 1) *key_data_type*, a data type of the key, 2) *value_data_type,* a data type of the value, and 3) *N*, the size of data sets to generate. In the algorithm, it generates the key data by calling *GenerateVariousDataTypes*(Int) and the value data by calling *GenerateVariousDataTypes*(String). For integer data, we generate a unique identifier by increasing it incrementally. For string data, we generate a string within a specific range of *MIN_LEN* and *MAX_LEN*. The data generation will be repeated until the size of data set becomes *N*. Using Algorithm 3, we can generate the synthetic data set with a variable size. As a result, we generated four actual synthetic data sets as presented in Table 4. Here, we use 4 as *MIN_LEN* and 100 as *MAX_LEN* and vary *N,* the size of data set we want to generate (Figs. 1 and 2).

### 2.3 How to utilize the acquired key-value data into key-value stores

The presented key-value data sets can be utilized for benchmarks to compare the performance of key-value stores in terms of two perspectives: 1) various data types and 2) various data set sizes. First, the most important reason why the key-value store is useful in big data applications is that it can support arbitrary data types by mapping any data type into the value part of the key-value pair. Thus, it is significant to support various data types to check the performance variation of key-value stores. Second, all the data storages are needed to prove their stable performance as the size of data sets is increased to show its scalability (Fig. 3).

**Fig. 4:**
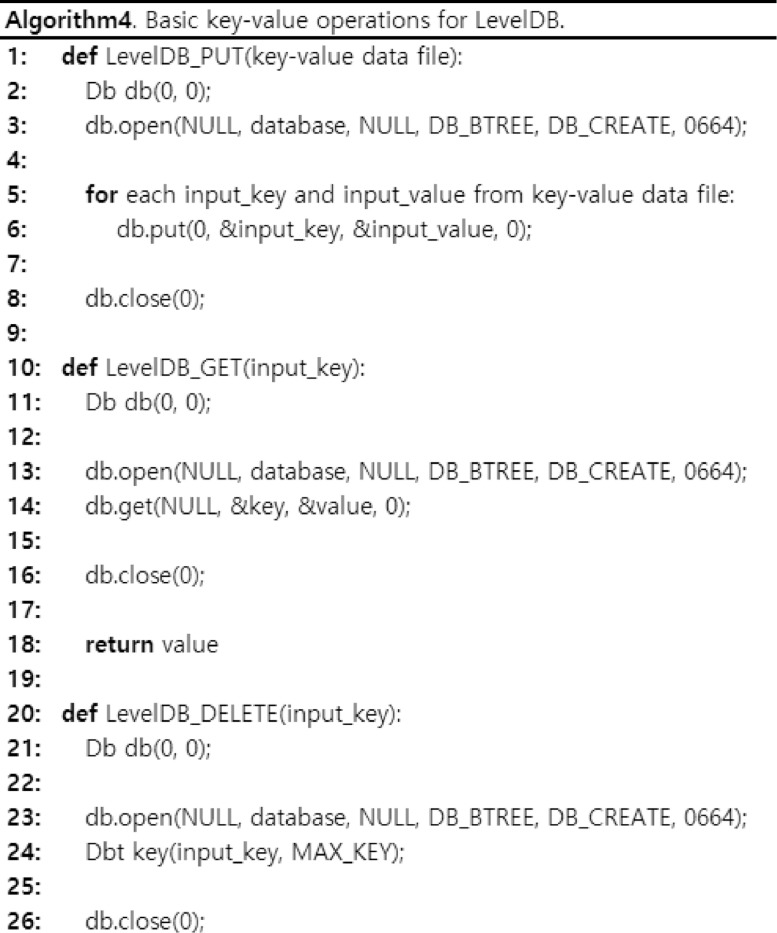
The algorithm of basic key-value operations for LevelDB.

In a research article [5], the key-value data sets presented in Table 2 and Table 4 were actually used to be stored in the well-known key-value stores: LevelDB [1], RocksDB [2], and Berkeley DB [3]. Then, their performances were extensively compared for the basic operations of the key-value stores: GET, PUT, and DELETE. Fig. 4 shows an example of basic key-value operations for LevelDB to show that the generated or crawled key-value data sets are actually used in the key-value stores. In the *LevelDB_PUT*() function, it receives the key-value data file, which can be one of data sets in Table 2 and Table 4, as the input. Then, it extracts each key-value pair and puts it into the key-value store. In the *LevelDB_GET*() function, it receives the *input_key* as the input to search a key-value pair. Then, it will find a key-value pair for a given *input_key*. For example, if we give the user ID for the data set *USER-FOLLOWERS*, then the *LEVELDB_GET*() function will return the number of followers for the user ID. In the *LevelDB_DELETE*() function, it receives the *input_key* as the input to delete a key-value pair. Then, it will delete a key-value pair for the given *input_key* from the key-value store. For RocksDB and BerkelyDB, we implemented all the basic operations for the key-value store in a similar way as in Fig. 4.

## Appendix Supplementary materials

Image, application 1

## Footnotes

1 https://developer.twitter.com/en/docs/tweets/data-dictionary/overview/tweet-object

2 https://developer.twitter.com/en/docs/basics/things-every-developer-should-know

3 Except for search keywords, we can use other conditions to filter Tweets such as since and until. In this algorithm, we use only the required condition, i.e., search keywords. For the other conditions, please refer to Tweepy document [4].
